# Downregulation of the L-PGDS/15d-PGJ2 Pathway Is Associated with Manganese-Induced Neuroinflammation and Cognitive Impairment Involving C/EBPβ and MMP9

**DOI:** 10.3390/antiox15080957

**Published:** 2026-07-31

**Authors:** Junrou Zhang, Kai Xu, Yan Ye, Junxiang Ma, Li Chen, Tian Chen, Piye Niu

**Affiliations:** 1 Beijing Key Laboratory of Environment and Aging, School of Public Health, Capital Medical University, Beijing 100069, China; 2 Department of Occupational Health, Beijing Center for Disease Control and Prevention, Beijing 100020, China

**Keywords:** cognitive function, inflammation, manganese, 15d-PGJ2

## Abstract

Excessive manganese (Mn) exposure is associated with neuroinflammation and cognitive impairment, yet the contribution of endogenous pro-resolving pathways to Mn neurotoxicity remains incompletely understood. Here, we examined the involvement of the lipocalin-type prostaglandin D synthase (L-PGDS)/15-deoxy-Δ^12,14^-prostaglandin J2 (15d-PGJ2) pathway using Mn-treated male C57BL/6J mice, BV2 microglia, and SH-SY5Y neurons, together with data from occupationally Mn-exposed workers (cross-sectional, hypothesis-generating). In the occupational cohort, circulating 15d-PGJ2 levels and L-PGDS expression were reduced following Mn exposure, consistent with the experimental findings. PPI network analysis and promoter prediction highlighted C/EBPβ and MMP9 as potential mediators of Mn-induced neuroinflammatory responses. Consistent with these observations, Mn exposure increased C/EBPβ and MMP9 expression, promoted M1 microglial polarization, elevated ROS production, and aggravated neuronal injury in a microglia–neuron co-culture system. Knocking down either C/EBPβ or MMP9 attenuated inflammatory activation and oxidative stress and reduced neuronal damage. Administration of exogenous 15d-PGJ2 suppressed Mn-induced increases in C/EBPβ and MMP9, alleviated inflammatory and oxidative responses, and partially ameliorated cognitive impairment in Mn-exposed mice. Transcriptomic analyses further showed that 15d-PGJ2 counteracted a substantial proportion of Mn-induced transcriptional alterations, particularly those related to inflammatory and neurodevelopmental processes. In addition, Ptgds knockdown reduced endogenous 15d-PGJ2 production and increased MMP9 expression, whereas exogenous 15d-PGJ2 largely reversed these changes. Together, these findings support the involvement of L-PGDS/15d-PGJ2 pathway dysregulation in Mn-induced neuroinflammation and suggest that restoration of 15d-PGJ2 signaling may represent a potential therapeutic approach for Mn-associated neurotoxicity.

## 1. Introduction

Manganese (Mn) is an essential trace element [1], but excessive occupational exposure can damage the central nervous system [2]. Epidemiological studies have consistently linked elevated Mn exposure to cognitive impairment, including deficits in learning, memory, and executive function [3]. Workers in mining, smelting, and steel production are particularly at risk [4]. Despite this, the cellular and molecular mechanisms behind Mn-induced cognitive decline remain incompletely understood.

In the complex process of Mn-induced neurotoxicity, neuroinflammation has been identified as a central mechanism associated with neuronal damage and dysfunction [5,6]. As resident immune cells of the central nervous system, microglia are the primary mediators of manganese-induced neuroinflammatory responses [7,8]. Upon Mn exposure, activated microglia release pro-inflammatory cytokines, including TNF-α and IL-1β, as well as reactive oxygen species (ROS) [9,10]. These inflammatory mediators can disrupt synaptic plasticity, impair neuronal function, and ultimately contribute to cognitive decline [11,12,13,14]. However, inflammation is not just about the strength of pro-inflammatory signals. Its resolution also depends on the timely action of endogenous anti-inflammatory and pro-resolving systems [15,16].

Arachidonic acid (AA) metabolism is an important regulatory network [17]. Among the bioactive lipid mediators generated through AA metabolism, 15-deoxy-Δ^12,14^-prostaglandin J2 (15d-PGJ2) has attracted considerable attention because of its potent anti-inflammatory and pro-resolving properties [18,19]. 15d-PGJ2 is an endogenous ligand of PPARγ and can inhibit NF-κB signaling [20,21]. In animal models of Alzheimer’s disease (AD) and cerebral ischemia, exogenous 15d-PGJ2 has been shown to reduce neuroinflammation and improve functional outcomes [22,23]. These findings suggest that 15d-PGJ2 may play an important neuroprotective role across a range of neuroinflammatory conditions.

In our previously published work [24], we found that Mn-exposed workers and Mn-treated mice both showed decreased levels of 15d-PGJ2 and its synthesizing enzyme L-PGDS. These changes correlated with cognitive decline and markers of neuroinflammation. Similar patterns were observed in human serum and the mouse striatum. These findings suggest that disruption of the L-PGDS/15d-PGJ2 pathway may be involved in Mn-induced neurotoxicity. However, the causal contribution of reduced endogenous 15d-PGJ2 to Mn-induced neuroinflammation and cognitive dysfunction, as well as the downstream molecular mechanisms involved, remains unclear. To address these gaps, we first used bioinformatics to screen for candidate molecules that might link 15d-PGJ2 reduction to neuroinflammation. We then validated the expression changes in animal and cell models, performed gene knockdown and co-culture experiments, and finally tested whether prophylactic intranasal administration of 15d-PGJ2 could mitigate cognitive and pathological changes in Mn-exposed mice.

We hypothesized that disruption of the L-PGDS/15d-PGJ2 pathway contributes to Mn-induced neuroinflammation and cognitive dysfunction through specific downstream inflammatory signaling mechanisms. To test this hypothesis, we combined bioinformatic analyses with human, animal, and cellular studies to identify potential mediators linking reduced 15d-PGJ2 to neuroinflammatory responses. We further investigated the involvement of the C/EBPβ–MMP9 signaling axis using gene knockdown approaches and a microglia–neuron co-culture model. Finally, we evaluated whether restoration of 15d-PGJ2 signaling through prophylactic intranasal administration could attenuate Mn-induced neuroinflammation and cognitive impairment.

## 2. Materials and Methods

### 2.1. Human Study Design and Participants

Some of the human cognitive assessments and inflammatory biomarker data have been published previously [24]. In brief, the study included 65 workers exposed to manganese and 106 controls (office workers from the same mine). For the purposes of this study, PTGDS scores were re-assessed in this cohort. All regression models were adjusted for age, sex, educational level, BMI, smoking, alcohol consumption, and hypertension status.

### 2.2. Identification of Potential Target Genes

Four gene sets relevant to Mn-induced neuroinflammation and cognitive dysfunction were collected: Mn-associated genes from CTD, neuroinflammation- and cognition-related genes from GeneCards, and differentially expressed genes from Mn-exposed mouse striatum (converted to human orthologs). The intersecting genes were considered candidate targets potentially involved in Mn-induced cognitive impairment and neuroinflammation. A PPI network was built with STRING and analyzed in Cytoscape 3.10.2 using the MCC algorithm (CytoHubba). The top 10 genes ranked by MCC scores were defined as hub genes for subsequent analyses. To predict C/EBPβ binding sites in the MMP9 promoter, the JASPAR matrix MA0466.2 was used. The promoter sequence (from −2000 bp to +200 bp of TSS) was retrieved from UCSC Genome Browser (GRCh38/hg38). Sites with a relative score ≥ 0.85 and *p* < 0.05 were retained. Positions, core sequences, and scores were recorded. A schematic was drawn with Adobe Illustrator 2023, marking the two high-score sites (−445 bp and −167 bp).

### 2.3. Gene Knockdown in BV2 Cells

**Figure 4:**
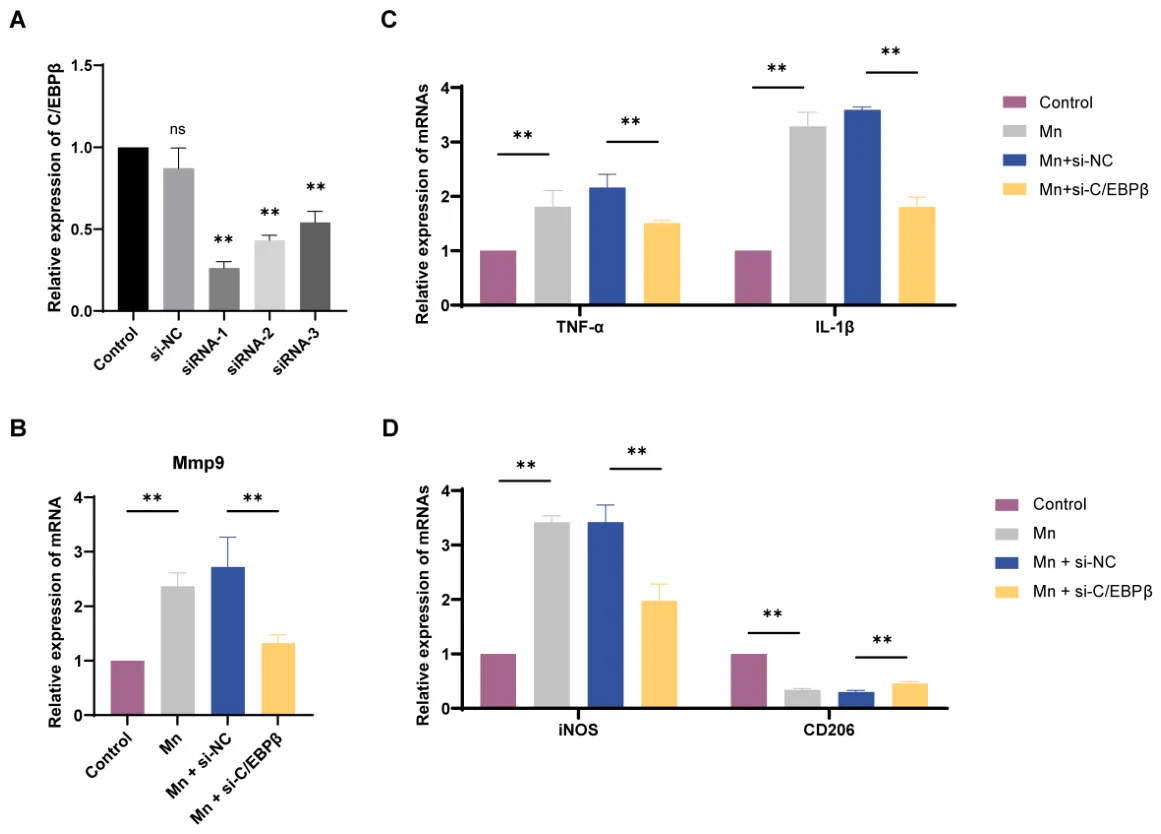
Inhibitory effect of *Cebpb* knockdown on Mn-induced *Mmp9* expression and inflammatory response. (**A**) qPCR analysis of Cebpb knockdown efficiency following siRNA transfection. (**B**) Effect of *Cebpb* knockdown on Mn-induced *Mmp9* mRNA expression determined by qPCR. (**C**) Effect of *Cebpb* knockdown on Mn-induced *TNF-α* and *IL-1β* release. (**D**) qPCR analysis of the effect of *Cebpb* knockdown on Mn-induced mRNA expression of M1 (*iNOS*) and M2 (*CD206*) polarization markers. Brackets indicate significant differences between indicated groups. Statistical analysis was performed using one-way ANOVA with Tukey’s post hoc test. (*n* = 3 independent experiments). ns, not significant, ** *p* < 0.01.

**Figure 6:**
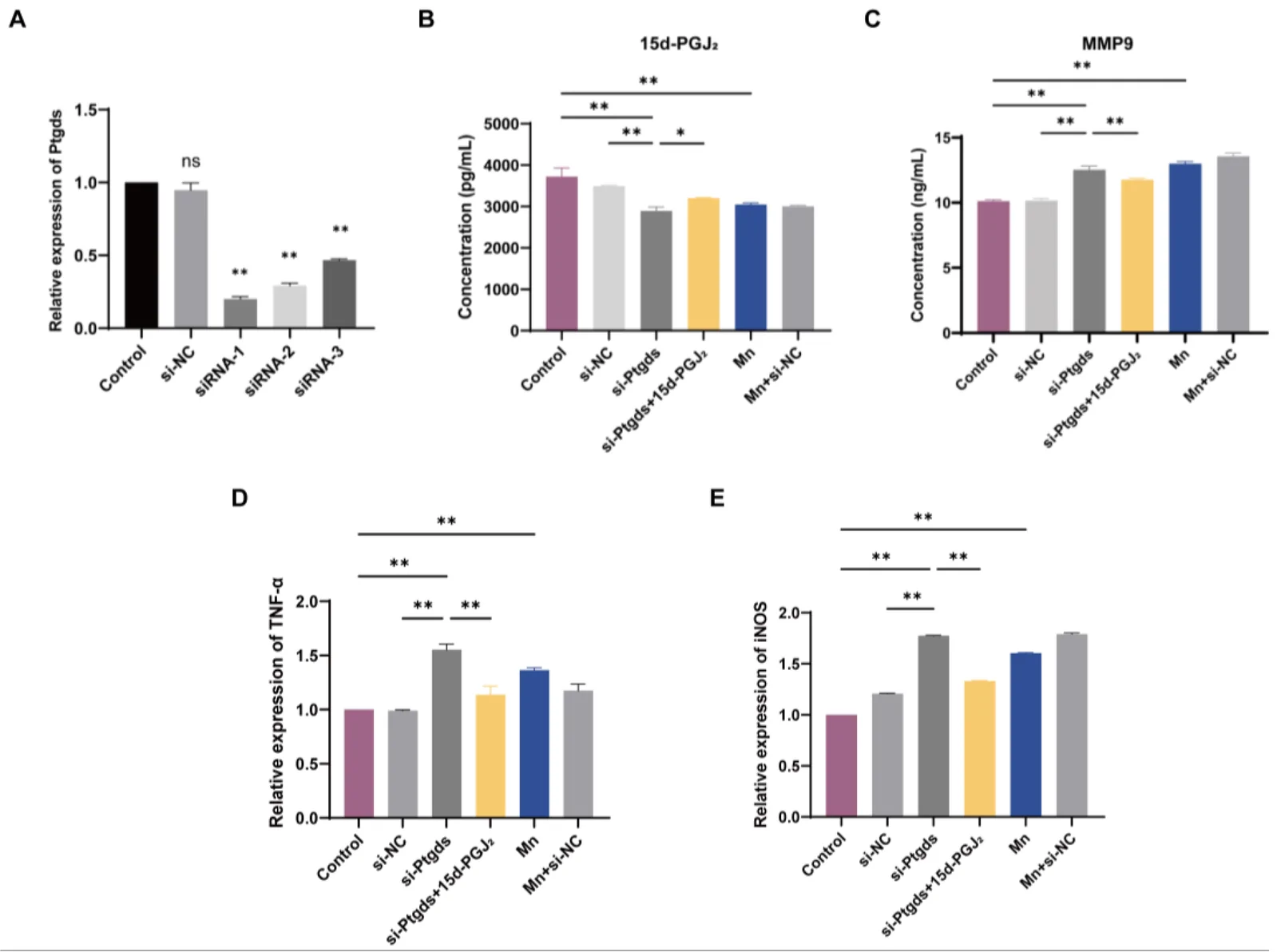
Effects of Ptgds knockdown on 15d-PGJ2 production, MMP9 expression, and microglial inflammatory responses. (**A**) *Ptgds* siRNA knockdown efficiency detected by qPCR; (**B**) 15d-PGJ2 levels in cells detected by ELISA; (**C**) MMP9 protein levels in cells detected by ELISA; (**D**) *TNF-α* mRNA levels detected by qPCR; (**E**) *iNOS* mRNA levels detected by qPCR. Control, untreated cells; si-NC, negative control siRNA; si-*Ptgds*, *Ptgds* siRNA; si-*Ptgds* + 15d-PGJ2, *Ptgds* siRNA with 15d-PGJ2; Mn, MnCl_2_ treatment; Mn + si-NC, negative control siRNA followed by MnCl_2_ treatment. (*n* = 3 independent experiments). * *p* < 0.05, ** *p* < 0.01.

Small interfering RNAs (siRNAs) targeting mouse *Ptgds, Mmp9, Cebpb*, or a negative control (si-NC) were used. BV2 cells were seeded into 6-well plates and transfected with 50 nM of each siRNA using Lipofectamine RNAiMAX (Invitrogen, Carlsbad, CA, USA)) according to the manufacturer’s protocol. After 48 h, knockdown efficiency was confirmed by quantitative real-time PCR (qPCR); the efficiencies for *Cebpb* and *Ptgds* are shown in Figure 4A and Figure 6A, respectively. For *Mmp9*, the siRNA sequence and knockdown efficiency (approximately 74%) were as previously described, using the same siRNA supplier and catalog number as in that study. For subsequent experiments, transfected cells were treated with MnCl_2_ (200 μmol/L, 6 h) or with 15d-PGJ2 (5 μmol/L, 1 h) before Mn exposure.

### 2.4. Cell Culture and Co-Culture

BV2 microglia were cultured in MEM with 10% FBS and 1% penicillin-streptomycin at 37 °C with 5% CO_2_. Cells were treated with MnCl_2_ (50–200 μmol/L) for 6 h, or pretreated with 5 μmol/L 15d-PGJ2 for 1 h before MnCl_2_ (200 μmol/L).

For the co-culture, BV2 cells were seeded in 6-well plates, then transfected with si-NC or si-*Mmp9*. After 24 h, they were exposed to 200 μmol/L MnCl_2_ for another 6 h. Supernatants were collected and centrifuged to obtain conditioned medium (CM). SH-SY5Y cells were subsequently cultured in a 1:1 mixture of fresh medium and the corresponding conditioned medium (CM). After 12 h, SH-SY5Y cells were harvested for subsequent molecular analyses.

### 2.5. Enzyme-Linked Immunosorbent Assay

Intracellular concentrations of 15d-PGJ2 and MMP9 were quantified using commercially available ELISA kits (Lunchangshuo Biotech, Xiamen, China). Absorbance was read at 450 nm on a microplate reader.

### 2.6. Measurement of Intracellular ROS

Intracellular ROS levels were measured using a DCFH-DA kit (KeyGEN BioTECH, Nanjing, China). BV2 cells were seeded in 6-well plates and cultured for 24 h. After treatment with MnCl_2_, cells were collected by gentle trypsinization, washed with PBS, and resuspended in serum-free DMEM containing 10 μmol/L DCFH-DA. The cells were incubated for 20 min at 37 °C in the dark, with gentle mixing every 3–5 min. The loading solution was then removed, and cells were washed twice with warm serum-free DMEM. Fluorescence intensity was measured immediately using a flow cytometer (Agilent Technologies, Santa Clara, CA, USA. excitation 488 nm, emission 525 nm). Data were analyzed as the mean fluorescence intensity relative to the control group.

### 2.7. Animals and Experimental Design

The animal study was approved by the Capital Medical University Animal Care Committee (AEEI-2021-212). Male C57BL/6J mice (8-wk, WT, naive) were randomly assigned to experimental groups. Randomization was performed using Excel’s RAND function, with allocation concealed from the experimenters. Mice were housed in an SPF facility, with food and water ad libitum, and without environmental enrichment.

Manganese exposure was induced using manganese(II) chloride tetrahydrate (MnCl_2_·4H_2_O; Sigma Life Science, St. Louis, MO, USA; Cat. No. 63535-250G-F; purity ≥ 99.0%, BioUltra grade). MnCl_2_ was selected because it is water-soluble, permits accurate and reproducible dosing, and is widely used in experimental studies of manganese neurotoxicity, thereby facilitating comparison with previous studies. 15-Deoxy-Δ^12,14^-prostaglandin J2 (15d-PGJ2; Sigma-Aldrich, St. Louis, MO, USA. Cat. No. 538927) was used for pharmacological intervention. Phosphate-buffered saline (PBS; Procell, Wuhan, China. Cat. No. PB180327) served as the vehicle control. All compounds were administered intranasally once daily, 5 days per week, for 12 weeks.

The animal experiments were conducted in two phases. In Phase 1 (*n* = 8 per group; data published previously), mice were assigned to control, low-dose Mn (2 mg/kg MnCl_2_), and high-dose Mn (20 mg/kg MnCl_2_) groups. The low dose was selected with reference to the occupational exposure limit specified in GBZ 2.1-2019 and converted to an experimental mouse dose after accounting for manganese content and interspecies scaling. The high dose was set at 10 times the low dose to evaluate dose-dependent effects of Mn exposure. Based on the findings from Phase 1, the high-dose regimen was selected for subsequent intervention studies.

In Phase 2, mice were assigned to four groups (*n* = 8 per group): control, Mn (20 mg/kg MnCl_2_), 15d-PGJ2 (300 ng/mouse), and Mn + 15d-PGJ2. In the combined treatment group, 15d-PGJ2 was administered 1 h before Mn exposure. Following the exposure period, behavioral assessments were performed, after which striatal tissue and serum samples were collected for subsequent biochemical and molecular analyses.

To minimize order effects, the testing sequence was alternated across groups, and cage positions on the racks were rotated weekly. Behavioral and biochemical assessors were blinded to group allocation. No animals were excluded; all mice completed the protocol, and no severe adverse events occurred. Humane endpoints (including weight loss > 20%, inability to eat or drink, and severe neurological signs) were monitored twice daily; none were reached, and therefore no early termination was needed. The statistician was not blinded during data analysis.

### 2.8. Behavioral Tests

Behavioral tests were performed in the order: rotarod, Y-maze, and Barnes maze, with 24 h intervals.

#### 2.8.1. Rotarod Test

Motor coordination and balance were assessed using an automated rotarod apparatus. Mice were acclimated to the testing room for 30 min. The rod was cleaned with 75% ethanol between animals. Latency to fall was recorded as the primary outcome measure.

#### 2.8.2. Y-Maze Test

Spontaneous alternation behavior in the Y-maze was used to assess spatial working memory [25] (three arms, each 30 cm long, 6 cm wide, 15 cm high). After a 30 min acclimation, each mouse was placed in the central area and allowed to explore freely for 8 min. Arm entries were defined as the entry of all four paws into an arm and were recorded throughout the test period. Spontaneous alternation rate was calculated as ((actual alternations)/(total entries-2)) × 100%.

#### 2.8.3. Barnes Maze Test

Spatial learning and memory were evaluated with the Barnes maze [26]. On day 1, a habituation trial allowed the mouse to find the target box (5 min max). During the acquisition phase (days 2–4), mice underwent two training trials per day, and the latency to locate the target box was recorded. On day 5 (probe test), the target box was removed; the latency to reach the former target location was recorded. The maze surface was cleaned with 75% ethanol between trials to eliminate olfactory cues.

### 2.9. Oxidative Stress Assays

Striatum tissues were homogenized in ice-cold Normal Saline (1:10, *w*/*v*) and centrifuged. Protein concentrations were determined using a bicinchoninic acid (BCA) assay kit. MDA levels were measured using the thiobarbituric acid (TBA) method (Beyotime, Shanghai, China. S0131S), and GSH content was determined using a colorimetric assay kit (Nanjing Jiancheng, Nanjing, China. A006-2-1). SOD activity was measured using a WST-8-based assay kit (Beyotime, S0101S), and hydroxyl radical scavenging capacity was determined using a Fenton reaction system kit (Nanjing Jiancheng, A018-1-1). All assays were performed according to the manufacturers’ instructions, and absorbance was read on a microplate reader. Results were normalized to protein content.

### 2.10. Real-Time RT-PCR

Total RNA was extracted from samples using TRIZOL reagent (Invitrogen, Carlsbad, CA, USA). Reverse transcription was carried out with the PrimeScript RT kit containing gDNA Eraser (TAKARA BIO INC, Shiga, Japan) to remove genomic DNA. Real-time PCR was performed using THUNDERBIRD SYBR qPCR Mix (Toyobo, Japan), following the manufacturer’s instructions. Relative mRNA expression levels were calculated using the 2^−ΔΔCt^ method, with Gapdh serving as the internal reference gene. Primer sequences are listed in Table S1.

### 2.11. Western Blotting Analysis

Striatal tissues or BV2 cells were lysed in RIPA buffer containing protease and phosphatase inhibitors. Protein concentration was determined by the BCA method. Equal amounts of protein were separated by SDS-PAGE and transferred onto PVDF membranes using a wet-transfer system. After blocking with 5% non-fat milk or 5% bovine serum albumin (BSA) for 1 h at room temperature, membranes were incubated overnight at 4 °C with primary antibodies against C/EBPβ (YT0553, ImmunoWay, 1:1000), p-C/EBPβ (YP0040, ImmunoWay, 1:1000), MMP9 (YM8311, ImmunoWay, 1:500), β-actin (YM8343, ImmunoWay, 1:8000) or Vinculin (CY5164, AbWays, 1:5000).

β-actin was used as the loading control for target proteins with molecular weights clearly different from β-actin (~42 kDa). When the molecular weight of the target protein was close to that of β-actin (e.g., C/EBPβ, ~36 kDa), Vinculin was used instead to avoid potential band overlap or interference during Western blot detection. After washing, the membranes were incubated with HRP-conjugated secondary antibodies for 1 h at room temperature. Protein bands were visualized using enhanced chemiluminescence (ECL). Band intensities were quantified using ImageJ v1.52i and normalized to the loading control.

### 2.12. RNA-Seq

Total RNA was extracted from striatal tissues of three mice per group. Total RNA quantity and purity were assessed using a Bioanalyzer 2100 and the RNA 6000 Nano LabChip Kit (Agilent, CA, USA, 5067-1511). A cDNA library was constructed from mouse striatum RNA. Sequencing was then performed on an Illumina Novaseq 6000 platform (LC-Bio Technology Co., Ltd., Hangzhou, China) following the manufacturer’s protocols. Paired-end reads (2 × 150 bp) were generated. Genes with *p* < 0.05 and fold change ≥ 1.2 were considered differentially expressed. A GO enrichment analysis was performed using DAVID on the differentially expressed genes that were significantly reversed following 15d-PGJ2 treatment. We also applied FDR-adjusted *p*-values (q < 0.05, Benjamini–Hochberg) with FC > 1.5; results are shown in Supplementary Figure S1.

### 2.13. UHPLC-MS/MS

UHPLC separation was performed on an ACQUITY Premier system (Waters, Milford, MA, USA.) using striatal tissues from five mice per group. Mass spectrometric detection was conducted using a SCIEX Triple Quad 6500+ system operated in multiple reaction monitoring (MRM) mode. Quantification was achieved by comparison with calibration curves generated using authentic standards.

### 2.14. Quantification and Statistical Analysis

All statistical analyses were carried out using R software (version 4.4.2). Normality was assessed using the Shapiro–Wilk test, and all datasets met the assumption of normal distribution. For two-group comparisons, Student’s *t*-test was used. For multigroup comparisons, one-way ANOVA followed by Tukey’s post hoc test was applied. Results are presented as mean ± SEM. Statistical significance was set at *p* < 0.05 and indicated as * *p* < 0.05, * *p* < 0.01. Graphs were prepared with GraphPad Prism (version 10.0).

## 3. Results

### 3.1. Bioinformatic Analysis Suggested C/EBPβ and MMP9 as Potential Key Molecules

**Figure 1:**
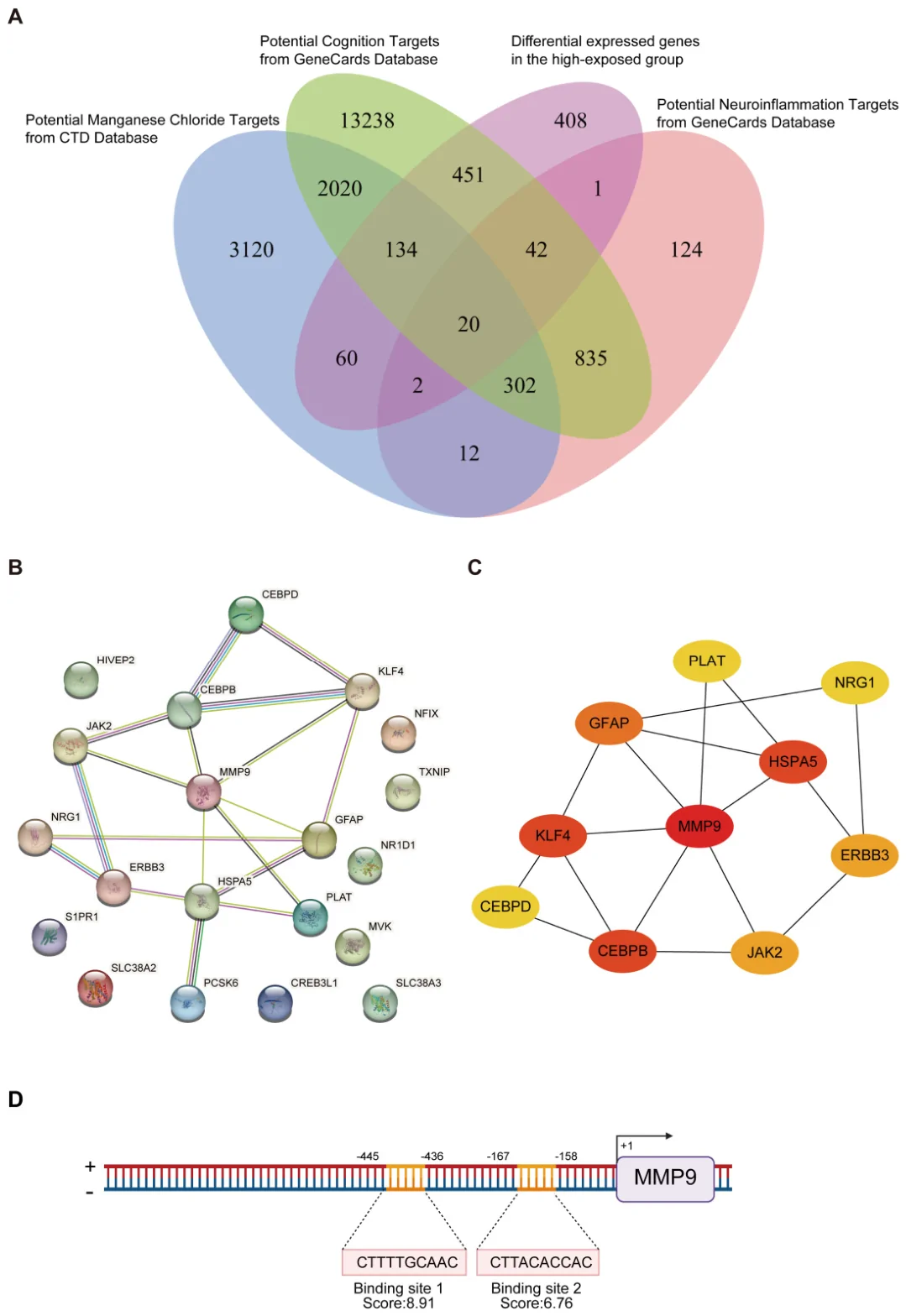
Identification of key genes in Mn neurotoxicity and prediction of C/EBPβ binding sites in the MMP9 promoter. (**A**) Venn diagram of CTD (*n* = 5670), GeneCards neuroinflammation (*n* = 1338), GeneCards cognition (*n* = 17,042), and transcriptomic (*n* = 1118) gene sets; 20 genes overlapped; (**B**) Protein-protein interaction network; (**C**) Top 10 hub genes (MCC ranking); MMP9 and C/EBPβ are highlighted; (**D**) Predicted C/EBPβ binding sites in the MMP9 promoter (position, score, and core sequence).

To identify candidate molecules potentially involved in Mn-induced neuroinflammation and cognitive impairment, we performed a bioinformatic analysis integrating four independent gene sets: Mn-related genes from the Comparative Toxicogenomics Database (CTD), genes associated with neuroinflammation and cognition from GeneCards, and differentially expressed genes from the striatum of Mn-exposed mice. The overlap among all four gene sets yielded 20 candidate genes (Figure 1A). We then built a protein–protein interaction (PPI) network using the STRING database (Figure 1B). To rank the nodes by their importance, we applied the Maximal Clique Centrality (MCC) algorithm in the CytoHubba plugin. MMP9 exhibited the highest MCC score among all hub genes, suggesting a potentially central role in the regulatory network. C/EBPβ was also ranked among the top hub genes identified by MCC analysis (Figure 1C). Based on their top rankings and biological relevance to inflammation, C/EBPβ and MMP9 were selected for further validation. The concurrent identification of MMP9 and C/EBPβ as hub genes raised the possibility of a regulatory relationship between these molecules. To investigate whether C/EBPβ may transcriptionally regulate MMP9 expression, we examined the promoter region of the MMP9 gene using the JASPAR database. The analysis predicted several potential binding sites for C/EBPβ. The two top-scoring sites were located at positions −445 bp and −167 bp upstream of the transcription start site, with relative scores of 0.9055 and 0.8764, respectively (Figure 1D). Both core binding sequences, 5′-CTTTTGCAAC-3′ and 5′-CTTACACCAC-3′, showed similarity to the reported C/EBPβ consensus binding motif. Notably, the −445 bp site was highly conserved across different mammalian species, indicating that this predicted binding site may warrant further investigation.

### 3.2. Mn Exposure Alters Expression of PTGDS, L-PGDS, C/EBPβ and MMP9

In our previous study, Mn exposure was associated with reduced serum 15d-PGJ2 levels in workers and dose-dependent decreases in striatal 15d-PGJ2 and L-PGDS expression in mice.

**Figure 2:**
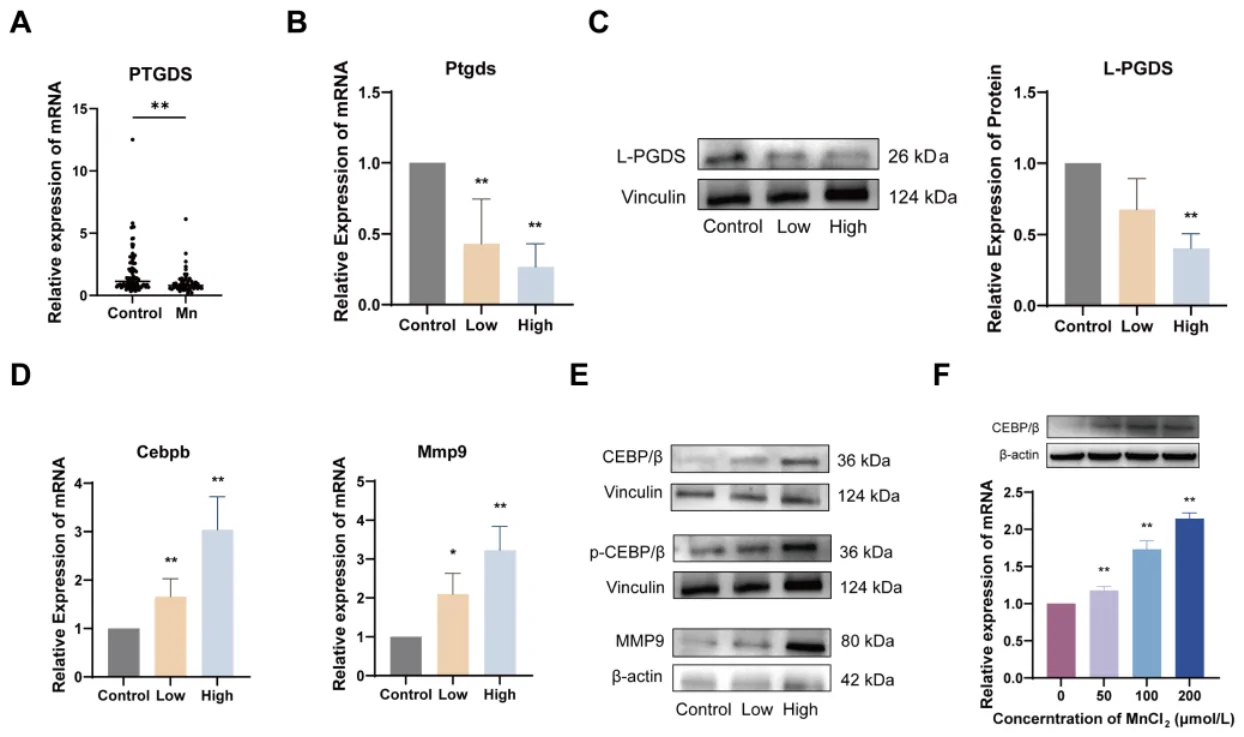
Effects of Mn exposure on PTGDS, C/EBPβ and MMP9 expression. Data are from Phase 1 animals. (**A**) PTGDS mRNA levels in human serum (qPCR); (**B**) *Ptgds* mRNA levels in mouse striatum; (**C**) Protein expression of L-PGDS in mouse striatum. (**D**) *Cebpb* and *Mmp9* mRNA levels in mouse striatum; (**E**) Protein expression of C/EBPβ, p-C/EBPβ, and Mmp9 in mouse striatum; (**F**) C/EBPβ levels in BV2. * *p* < 0.05, ** *p* < 0.01. Note: Panels (**C**,**E**) share the same Vinculin loading control, as both proteins were analyzed from the same biological samples under identical experimental conditions. Therefore, a common Vinculin blot is presented for both analyses. Representative blots from pooled striatal samples (*n* = 1 pool/group; 3 mice per pool).

To extend these observations, we further quantified *PTGDS* mRNA expression in peripheral blood samples obtained from the same occupational cohort. As shown in Figure 2A, *PTGDS* was significantly downregulated in the exposure group. The decrease in *PTGDS* expression in exposed workers was consistent with the reduction in L-PGDS expression observed in the mouse striatum (Figure 2B,C).

In striatal tissues from the same Mn-exposed mice, Western blot analysis demonstrated that Mn exposure significantly increased total C/EBPβ and phosphorylated C/EBPβ (p-C/EBPβ) protein levels in the striatum (Figure 2D). MMP9 protein expression was similarly elevated in Mn-treated mice compared with controls. qPCR confirmed that both *Cebpb* and *Mmp9* mRNAs were elevated in a dose-dependent manner (Figure 2D,E). Similarly, Mn exposure increased total C/EBPβ and MMP9 protein levels in BV2 microglial cells (Figure 2F). qPCR analysis further demonstrated that *Cebpb* and *Mmp9* mRNA expression was significantly elevated following Mn exposure. Collectively, these findings demonstrate that Mn exposure is associated with reduced PTGDS/L-PGDS expression and concomitant activation of the C/EBPβ–MMP9 pathway in both mouse striatum and BV2 cells.

### 3.3. Knockdown of MMP9 Attenuates Microglia-Mediated Neuronal Injury

Our previous study demonstrated that MMP9 knockdown attenuated Mn-induced oxidative stress and M1 polarization in BV2 microglia [24]. Building upon these findings, we next investigated whether microglial MMP9 contributes to subsequent neuronal injury.

To determine whether microglial MMP9 mediates indirect neurotoxicity, conditioned medium (CM) was collected from Mn-treated BV2 cells and subsequently transferred to SH-SY5Y neurons.

**Figure 3:**
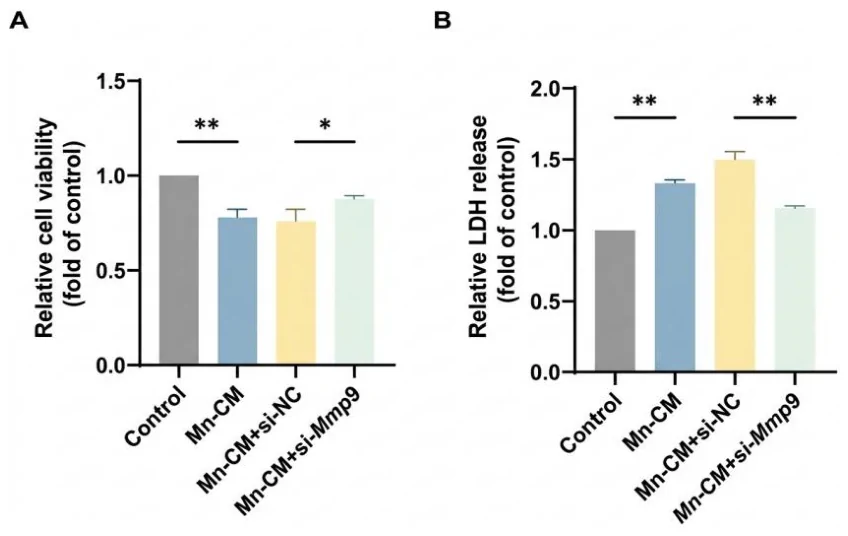
Knockdown of Mmp9 in BV2 microglia alleviates SH-SY5Y neuronal injury induced by Mn-treated conditioned medium (CM). (**A**) SH-SY5Y cell viability assessed by CCK-8 assay; (**B**) Neuronal cell membrane integrity determined by LDH release assay. Control: untreated control group; Mn-CM: conditioned medium from BV2 cells treated with Mn; Mn-CM + si-NC: conditioned medium from Mn-treated BV2 cells transfected with negative control siRNA; Mn-CM + si-*Mmp9*: conditioned medium from Mn-treated BV2 cells transfected with Mmp9 siRNA. (*n* = 3 independent experiments). * *p* < 0.05, ** *p* < 0.01.

As shown in Figure 3A, conditioned medium derived from Mn-treated BV2 cells significantly reduced SH-SY5Y cell viability compared with the control group, as determined by the CCK-8 assay (*p* < 0.01). Knocking down *Mmp9* in BV2 cells significantly restored neuronal viability compared with the Mn-CM + si-NC group (*p* < 0.05).

Consistent with these findings, LDH release was markedly increased in neurons exposed to Mn-CM, indicating enhanced membrane damage and neuronal injury (Figure 3B, *p* < 0.01). Importantly, conditioned medium collected from *Mmp9*-knockdown BV2 cells significantly reduced LDH release compared with the Mn-CM + si-NC group (*p* < 0.01); the marginal increase in the si-NC control is attributable to transfection stress and does not alter the key interpretation.

These results suggest that microglial MMP9 contributes to Mn-induced indirect neurotoxicity and that suppression of MMP9 alleviates neuronal injury mediated by activated microglia.

### 3.4. Knockdown of C/EBPβ Suppresses Mn-Induced MMP9 Upregulation and Inflammatory Responses

To investigate the role of C/EBPβ in Mn-induced inflammatory responses, Cebpb expression was knocked down in BV2 cells using siRNA. Among the three siRNAs tested, siRNA-1 showed the highest knockdown efficiency and was therefore selected for subsequent experiments (Figure 4A).

As shown in Figure 4B, Mn exposure significantly increased *Mmp9* mRNA expression compared with the control group. This effect was not altered by transfection with negative control siRNA. In contrast, *Cebpb* knockdown markedly attenuated Mn-induced *Mmp9* upregulation (*p* < 0.05), suggesting that C/EBPβ is involved in the transcriptional regulation of Mmp9.

Consistent with the changes in Mmp9 expression, Mn exposure significantly increased the expression of the pro-inflammatory cytokines TNF-α and IL-1β, whereas *Cebpb* knockdown markedly reduced their expression levels (Figure 4C).

Furthermore, *Cebpb* knockdown significantly decreased the expression of the M1 polarization marker iNOS and partially restored the expression of the M2 marker CD206 following Mn exposure (Figure 4D). These findings indicate that C/EBPβ contributes to Mn-induced microglial pro-inflammatory polarization.

Collectively, these results support a critical role for C/EBPβ in mediating Mn-induced Mmp9 expression and inflammatory activation in microglia.

### 3.5. Exogenous 15d-PGJ2 Suppresses Mn-Induced C/EBPβ Activation, and C/EBPβ Knockdown Enhances Its Anti-Inflammatory Effect

To determine whether C/EBPβ mediates the anti-inflammatory effect of 15d-PGJ2, BV2 cells were pretreated with 15d-PGJ2 with or without *Cebpb* siRNA, followed by Mn exposure.

**Figure 5:**
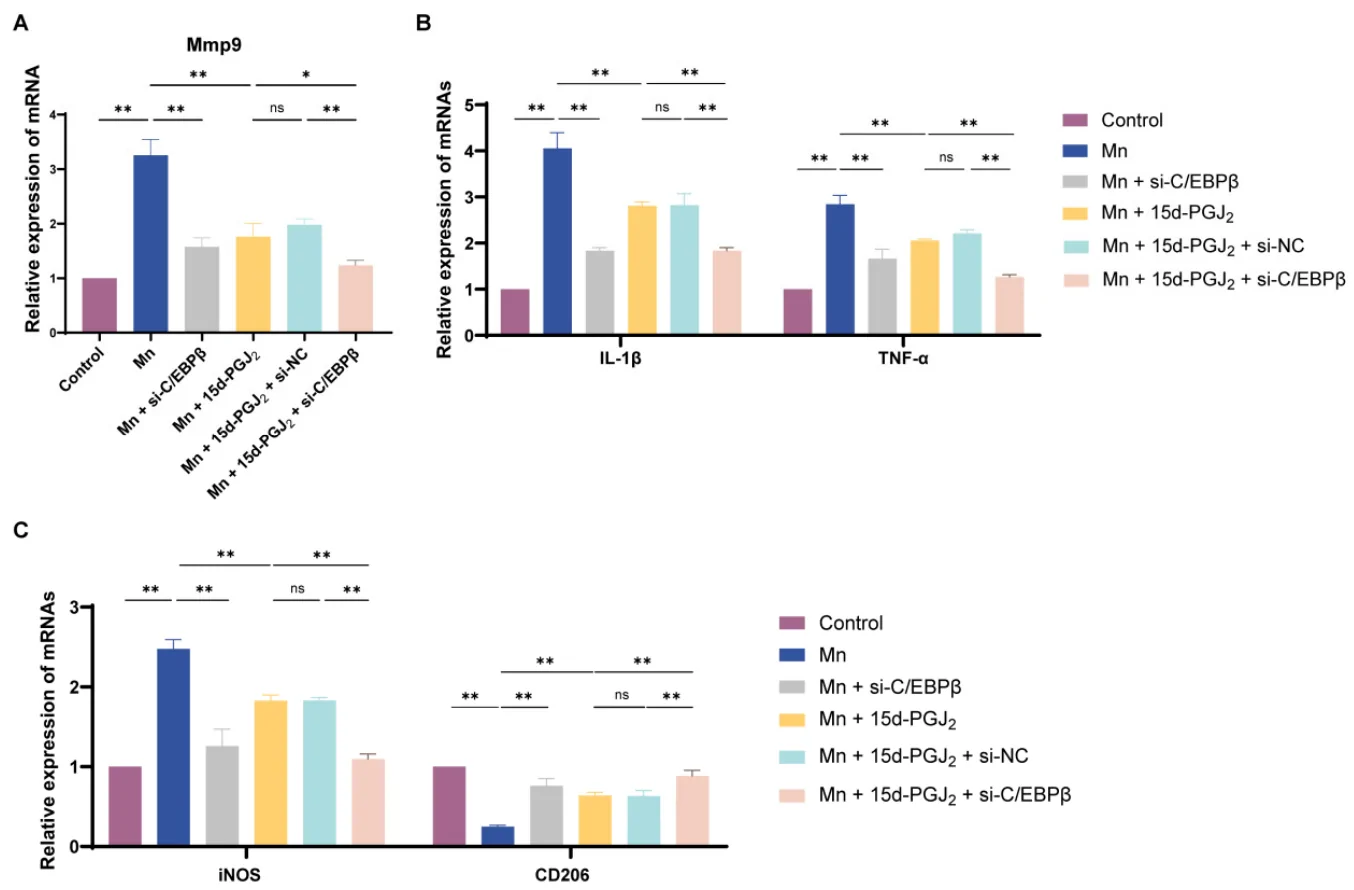
Knockdown of *Cebpb* enhances the inhibitory effect of 15d-PGJ_2_ on Mn-induced microglial inflammation. (**A**) *Mmp9* mRNA expression levels in BV2 cells; (**B**) TNF-α and IL-1β levels in BV2 cells; (**C**) mRNA expression levels of M1 polarization marker *iNOS* and M2 polarization marker *CD206* in BV2 cells. (*n* = 3 independent experiments). ns, not significant, * *p* < 0.05, ** *p* < 0.01.

As shown in Figure 5A, Mn exposure significantly increased *Mmp9* mRNA expression compared with the control group (*p* < 0.01). Both *Cebpb* knockdown and 15d-PGJ2 treatment significantly attenuated Mn-induced *Mmp9* upregulation. Notably, the combination of 15d-PGJ2 and *Cebpb* knockdown further reduced *Mmp9* expression compared with 15d-PGJ2 treatment alone (*p* < 0.01).

Similarly, Mn-induced increases in *IL-1β* and *TNF-α* expression were markedly suppressed by either *Cebpb* knockdown or 15d-PGJ2 treatment (Figure 5B, *p* < 0.01). Moreover, combined treatment produced a greater inhibitory effect than 15d-PGJ2 alone, indicating that suppression of C/EBPβ enhances the anti-inflammatory action of 15d-PGJ2.

Consistent with these findings, Mn exposure significantly increased the expression of the M1 polarization marker iNOS and decreased the expression of the M2 marker CD206 (Figure 5C). Both alterations were partially reversed by 15d-PGJ2 treatment or Cebpb knockdown. Importantly, combined treatment further reduced iNOS expression and restored CD206 expression compared with 15d-PGJ2 treatment alone.

Collectively, these findings suggest that C/EBPβ contributes to Mn-induced microglial inflammatory activation and that inhibition of C/EBPβ potentiates the anti-inflammatory effects of 15d-PGJ2.

### 3.6. Knockdown of Ptgds Reduces 15d-PGJ2 and Increases MMP9, Both of Which Are Reversed by Exogenous 15d-PGJ2

To determine whether endogenous 15d-PGJ2 regulates MMP9 expression, *Ptgds* was knocked down in BV2 cells. As shown in Figure 6A, *Ptgds* siRNA effectively reduced *Ptgds* mRNA levels compared with the control and si-NC groups (*p* < 0.01), confirming successful knockdown.

Consistent with the role of PTGDS in 15d-PGJ2 biosynthesis, *Ptgds* knockdown significantly decreased cellular 15d-PGJ2 levels (Figure 6B, *p* < 0.01), an effect comparable to that observed in the Mn group. Importantly, exogenous 15d-PGJ2 supplementation restored 15d-PGJ2 levels in Ptgds-deficient cells. Mn exposure alone also reduced 15d-PGJ2 levels, and this effect was not altered by si-NC transfection (Mn + si-NC vs. Mn, *p* > 0.05).

Concomitantly, *Ptgds* knockdown significantly increased MMP9 expression compared with the control group (Figure 6C, *p* < 0.01), phenocopying the effect observed with Mn exposure. Notably, treatment with exogenous 15d-PGJ2 markedly attenuated the increase in MMP9 upregulation induced by *Ptgds* knockdown (*p* < 0.01). Ptgds knockdown significantly upregulated *TNF-α* (Figure 6D), indicating an inflammatory response, and *iNOS* mRNA, indicating microglial polarization (Figure 6E). Both effects were reversed by exogenous 15d-PGJ2 supplementation (*p* < 0.01).

These findings indicate that PTGDS-derived 15d-PGJ2 negatively regulates MMP9 expression and suggest that reduced endogenous 15d-PGJ2 contributes to MMP9 upregulation under Mn-induced inflammatory conditions.

### 3.7. Prophylactic 15d-PGJ2 Administration Alleviates Mn-Induced Neurotoxicity and Neuroinflammation In Vivo

**Figure 7:**
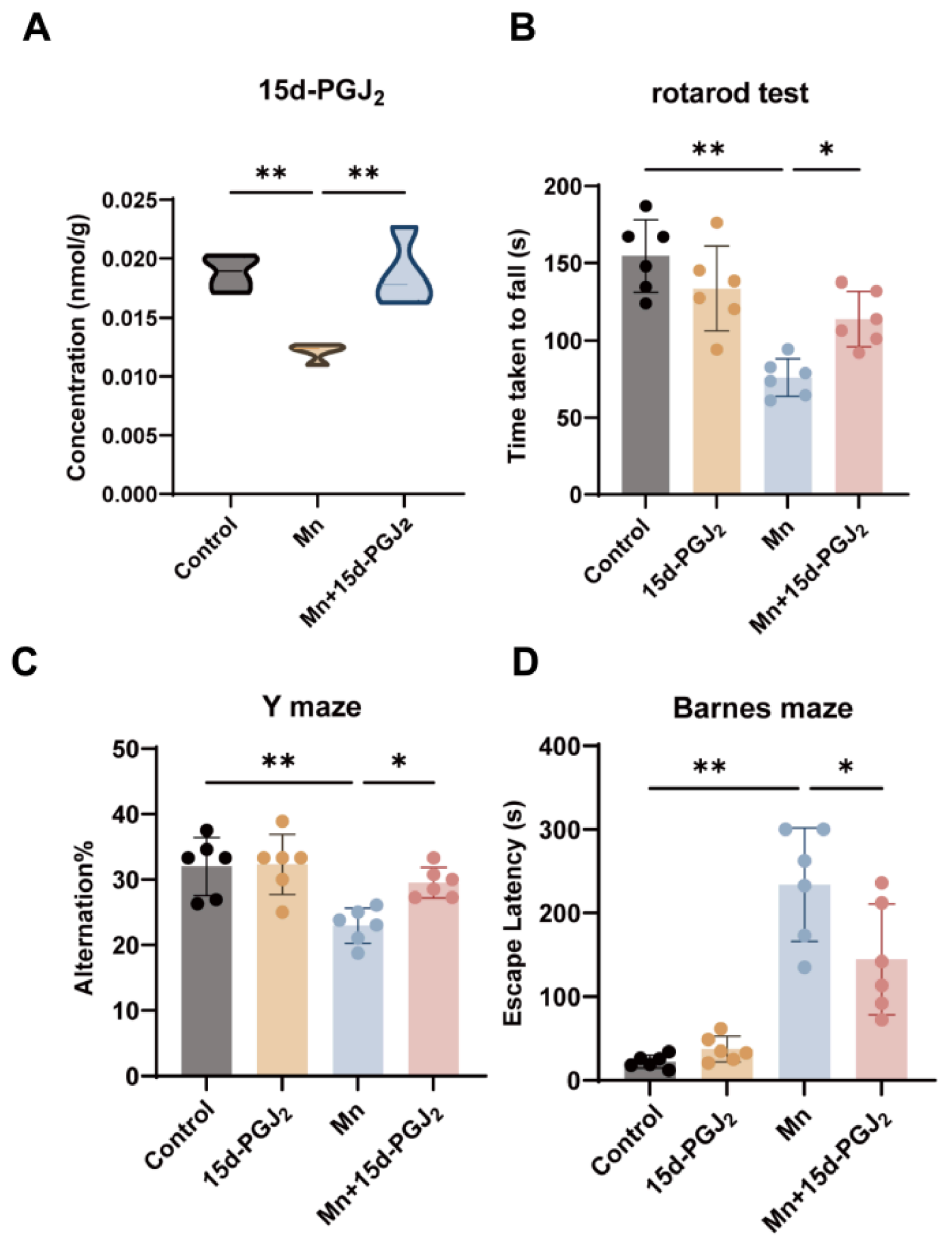
Effects of 15d-PGJ2 intervention on neurobehavior in Mn-exposed mice. (**A**) 15d-PGJ2 levels in striatal tissue; (**B**) Rotarod test; (**C**) Y-maze test; (**D**) Barnes maze test. (*n* = 6). ns, not significant, * *p* < 0.05, ** *p* < 0.01.

To evaluate the protective effects of 15d-PGJ2 in vivo, mice were prophylactically treated with 15d-PGJ2 prior to Mn exposure. As shown in Figure 7A, Mn exposure significantly reduced striatal 15d-PGJ2 levels, whereas administration of 15d-PGJ2 restored its levels. Behavioral assessments demonstrated that Mn exposure induced significant motor and cognitive deficits, as evidenced by impaired performance in the rotarod, Y-maze, and Barnes maze tests (Figure 7B–D). Pretreatment with 15d-PGJ2 markedly ameliorated these behavioral impairments, indicating a protective effect against Mn-induced neurological dysfunction.

**Figure 8:**
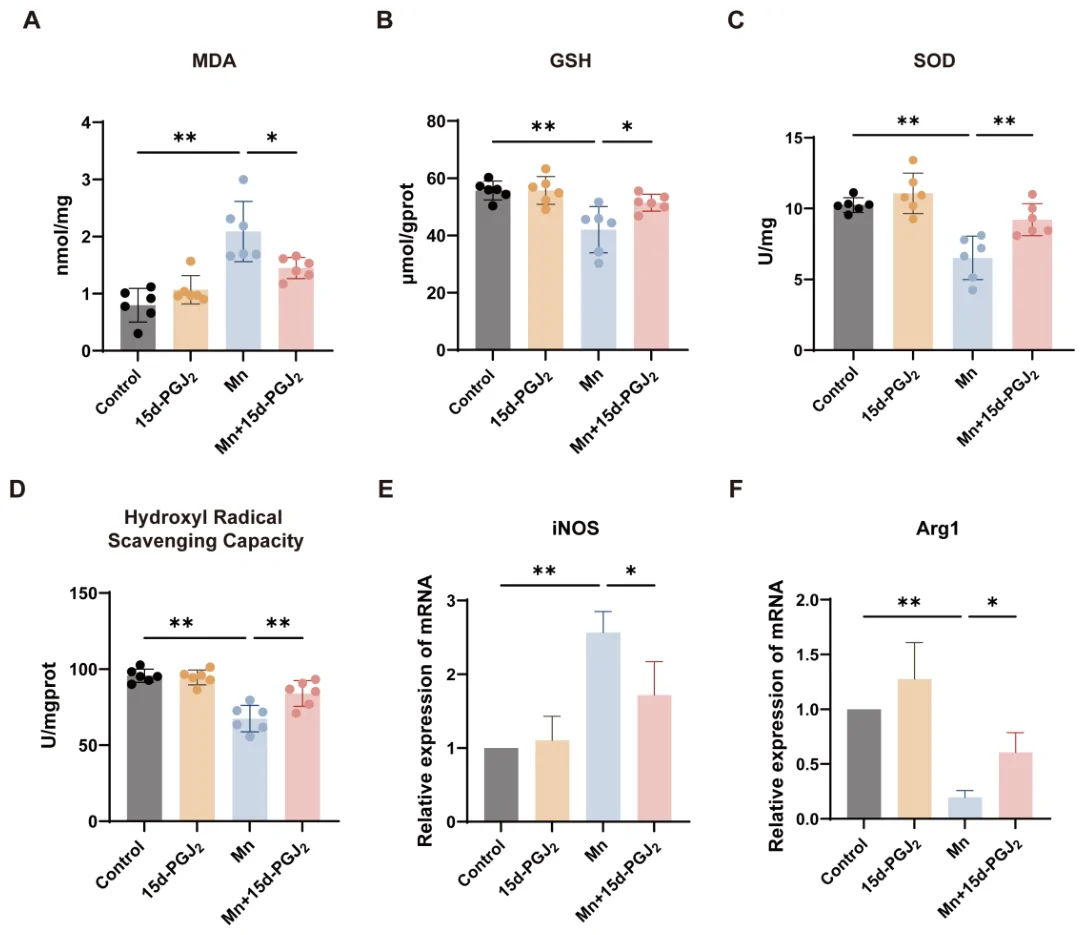
Effects of 15d-PGJ2 intervention on oxidative stress and microglial polarization in the striatum of Mn-exposed mice. (**A**) Malondialdehyde (MDA) content; (**B**) Glutathione (GSH) level; (**C**) Superoxide dismutase (SOD) activity; (**D**) Total antioxidant capacity (hydroxyl radical inhibition); (**E**) Relative mRNA expression of M1 marker inducible nitric oxide synthase (iNOS); (**F**) Relative mRNA expression of M2 marker Arg1. (*n* = 6). * *p* < 0.05, ** *p* < 0.01.

To further investigate the underlying mechanisms, oxidative stress and inflammatory markers were evaluated in striatal tissue. Mn exposure significantly increased malondialdehyde (MDA) levels while decreasing glutathione (GSH) content, superoxide dismutase (SOD) activity, and total antioxidant capacity (Figure 8A–D). These alterations were partially reversed by 15d-PGJ2 treatment, indicating attenuation of Mn-induced oxidative stress. In addition, Mn exposure significantly increased the expression of the M1 microglial marker iNOS and decreased the expression of the M2 marker Arg-1 (Figure 8E,F). Treatment with 15d-PGJ2 largely reversed these changes, suggesting suppression of pro-inflammatory microglial activation and promotion of an anti-inflammatory phenotype.

**Figure 9:**
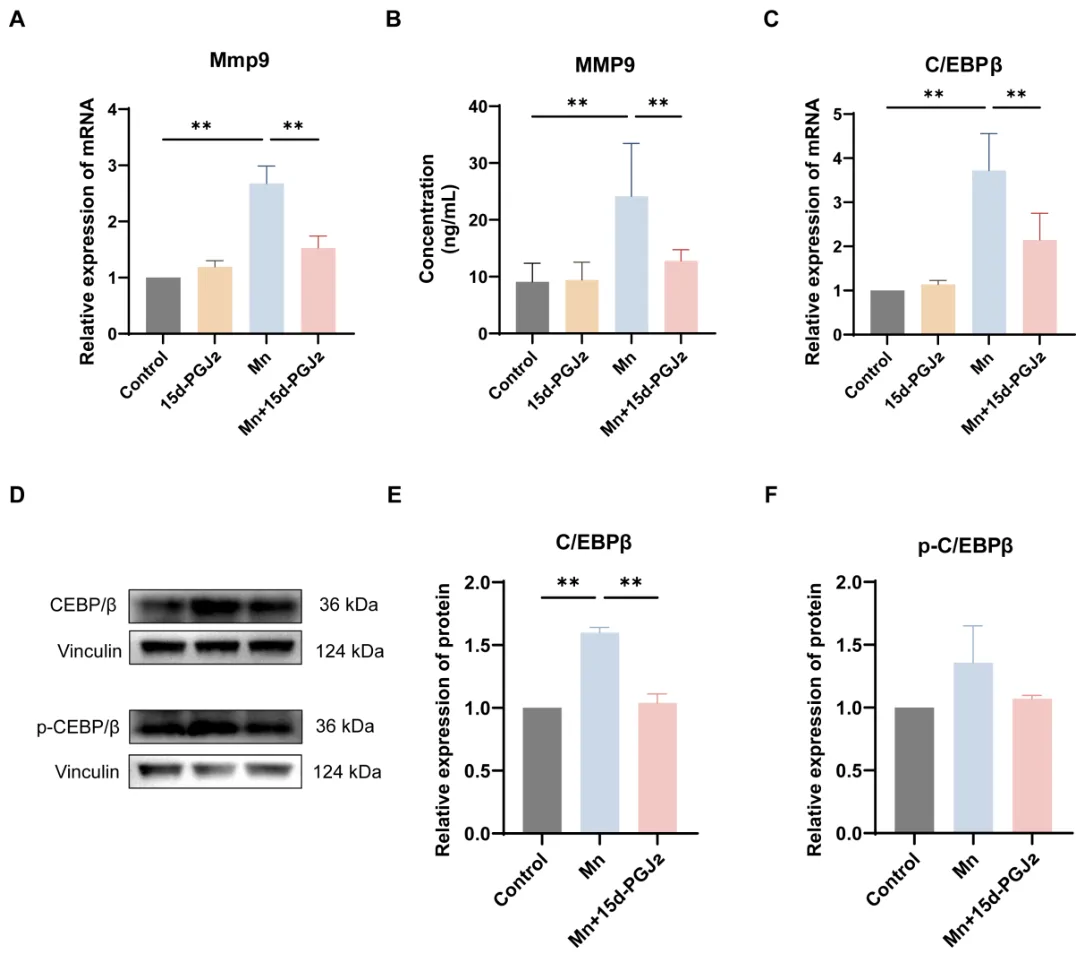
Effects of 15d-PGJ_2_ intervention on C/EBPβ and MMP9 expression in the striatum of Mn-exposed mice. (**A**) *Mmp9* mRNA expression detected by qPCR; (**B**) MMP9 protein levels detected by ELISA; (**C**) *Cebpb* mRNA expression detected by qPCR; (**D**) Representative Western blot bands of C/EBPβ and p-C/EBPβ protein expression, representative blots from pooled striatal samples (*n* = 1 pool/group; 3 mice per pool); (**E**) Semi-quantitative analysis of total C/EBPβ protein expression; (**F**) Semi-quantitative analysis of p-C/EBPβ protein expression. ** *p* < 0.01.

To validate the involvement of the C/EBPβ–MMP9 pathway in vivo, the expression of C/EBPβ and MMP9 was examined in the striatum. Mn exposure significantly increased both *Mmp9* mRNA expression and MMP9 protein levels, whereas these increases were markedly attenuated by 15d-PGJ2 treatment (Figure 9A,B). Similarly, Mn exposure elevated *Cebpb* mRNA expression and total C/EBPβ protein levels, which were significantly reduced following 15d-PGJ2 administration, while p-C/EBPβ showed a similar trend (Figure 9C–F). These findings are consistent with the in vitro results and support the involvement of the C/EBPβ–MMP9 signaling pathway in the neuroprotective effects of 15d-PGJ2.

**Figure 10:**
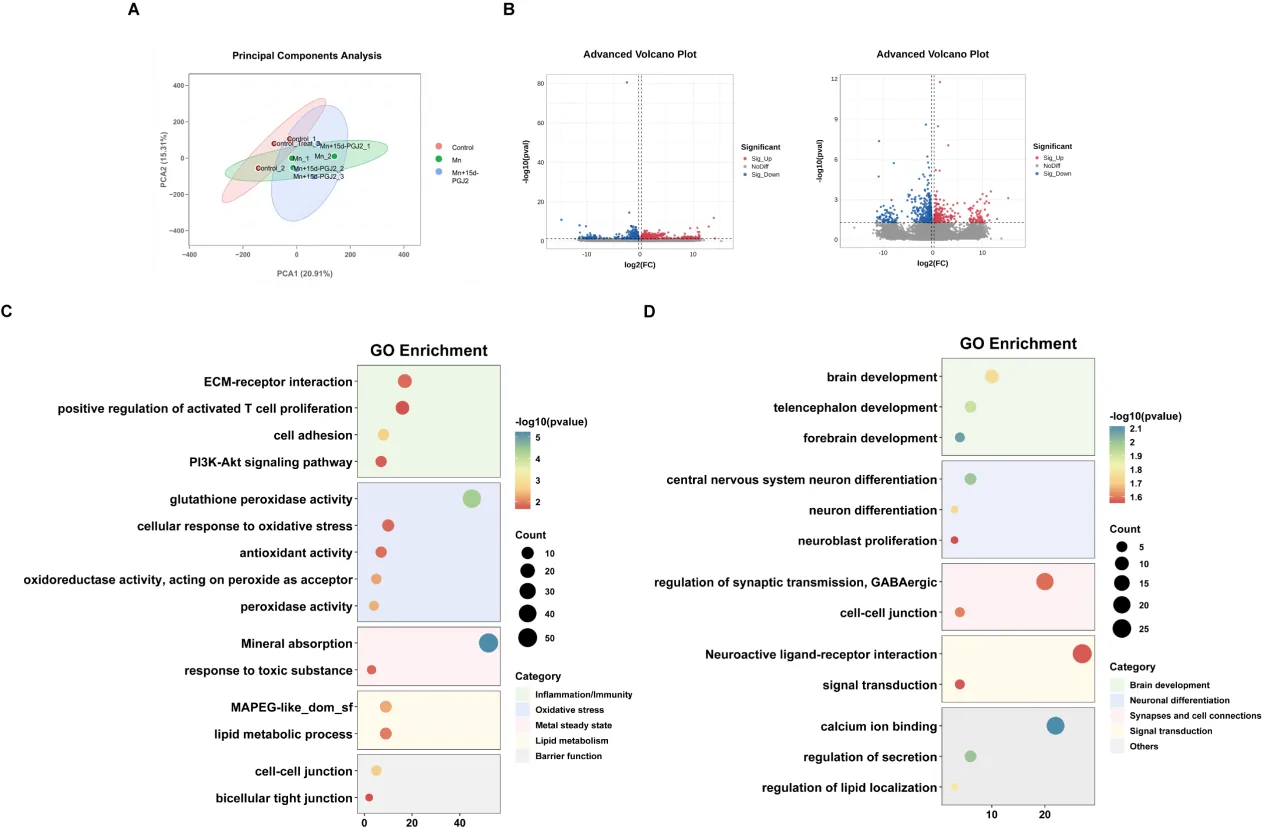
Transcriptomic analysis reveals the effects of 15d-PGJ2 intervention on gene expression profiles in the striatum of Mn-exposed mice. (**A**) Principal Component Analysis (PCA) score plot showing the distribution of samples from the control, Mn-exposed, and 15d-PGJ2 intervention groups; (**B**) volcano plot of differentially expressed genes; Volcano plot showing differentially expressed genes (FC > 1.2, *p* < 0.05). FDR-adjusted results (FC > 1.5, q < 0.05) are shown in Supplementary Figure S1. (**C**) Functional enrichment analysis of 107 genes that were upregulated by Mn exposure and significantly downregulated after intervention; (**D**) functional enrichment analysis of 61 genes that were downregulated by Mn exposure and significantly upregulated after intervention.

To further characterize the global transcriptional responses associated with 15d-PGJ2 treatment, RNA sequencing was performed on striatal tissues. Principal component analysis revealed a clear separation among the control, Mn-exposed, and 15d-PGJ2-treated groups, indicating distinct transcriptional profiles (Figure 10A). Differential expression analysis identified 836 upregulated and 944 downregulated genes in the Mn group compared with controls (fold change > 1.2, *p* < 0.05) (Figure 10B). Among the genes upregulated by Mn exposure, 107 were significantly downregulated following 15d-PGJ2 treatment and were primarily enriched in biological processes related to inflammatory responses, oxidative stress, and apoptosis (Figure 10C). Conversely, among the genes suppressed by Mn exposure, 61 were restored by 15d-PGJ2 treatment and were enriched in processes associated with forebrain development, neuronal differentiation, and synaptic transmission (Figure 10D). These transcriptomic alterations further support the notion that 15d-PGJ2 counteracts Mn-induced neurotoxicity. It does so by suppressing inflammatory and oxidative stress pathways while also restoring gene programs that are important for neuronal function and development.

## 4. Discussion

This study extends our previously published work [24]. In that earlier investigation, both occupational cohort data and animal experiments indicated that Mn exposure is associated with cognitive impairment and accompanied by a reduction in the endogenous anti-inflammatory mediator 15d-PGJ2. However, two questions remained: whether the decrease in 15d-PGJ2 actively contributes to downstream inflammatory events, and whether exogenous 15d-PGJ2 can provide protection. To address these, the present study integrated bioinformatics, gene knockdown, co-culture and prophylactic intervention approaches to explore potential downstream effectors of 15d-PGJ2 and their modulatory effects. The main observations include: C/EBPβ and MMP9 may represent downstream molecules involved in Mn-induced neuroinflammation; knockdown of either MMP9 or C/EBPβ mitigates microglial activation and neuronal injury; exogenous 15d-PGJ2 supplementation in vitro and in vivo reverses the Mn-associated molecular alterations and improves cognitive behaviour. Collectively, these findings suggest that decreased 15d-PGJ2, together with changes in the C/EBPβ-MMP9 cascade, may participate in Mn neurotoxicity, and that exogenous 15d-PGJ2 confers a certain degree of protective potential.

15d-PGJ2 is not synthesized directly. Instead, it arises from the non-enzymatic dehydration of PGD2. The synthesis of PGD2, in turn, requires the enzyme prostaglandin D synthase. This enzyme exists in two isoforms [27], namely the hematopoietic type (H-PGDS) and the lipocalin-type (L-PGDS). In the brain, L-PGDS is the main enzyme that produces PGD2 and subsequently 15d-PGJ2 [28]. It also helps maintain cerebral vascular homeostasis [29].

In this study, we observed that serum 15d-PGJ2 levels in Mn-exposed workers were lower than those in the control group. Simultaneously, the expression of PTGDS, the gene encoding L-PGDS, in their peripheral blood also showed a decreasing trend. This phenomenon was recapitulated in animal experiments. In Mn-exposed mice, striatal L-PGDS, at both mRNA and protein levels, decreased progressively with increasing exposure dose, exhibiting a clear dose–response relationship. Combining data from both humans and mice, a consistent conclusion can be drawn. Mn exposure is closely associated with reduced 15d-PGJ2 and downregulated L-PGDS expression.

15d-PGJ2 is an endogenous anti-inflammatory mediator derived from arachidonic acid metabolism. The decrease in 15d-PGJ2 observed after Mn exposure in our study may be directly linked to suppression of L-PGDS. L-PGDS is a key enzyme in brain PGD2 production and its conversion to 15d-PGJ2. Notably, the downregulation of PTGDS mRNA in exposed workers matched the trend seen in mice. This suggests possible cross-species consistency. The human data are cross-sectional and provide supportive background for the mechanistic findings, consistent with the animal results. Cross-sectional designs, however, have inherent limitations for causal inference, and confounding factors such as education level may also affect the estimates.

Bioinformatics analysis identified C/EBPβ and MMP9 as key nodes in the Mn neurotoxicity network, and promoter analysis suggested that MMP9 may contain a C/EBPβ binding site. C/EBPβ is a key transcription factor in inflammation. It regulates microglial activation, pro-inflammatory mediator expression, and neuronal damage in various neurological disease models, as shown in previous studies [30,31,32]. MMP9 is a zinc-dependent protease. As a key mediator of synaptic remodeling and neuroinflammation, its levels are closely associated with cognitive function [33]. Elevated MMP9 levels in peripheral blood can compromise blood–brain barrier integrity. This leads to infiltration of inflammatory factors into the central nervous system, which exacerbates neurological damage and cognitive impairment [34]. Targeted inhibition of MMP9 has been proposed as a therapeutic strategy in epilepsy research [35]. In Mn-treated mice and BV2 cells, C/EBPβ activation was associated with elevated MMP9 expression. Knockdown of MMP9 or C/EBPβ attenuated both microglial activation and neuronal damage. Co-culture experiments further indicated that MMP9 might mediate the neurotoxic effects exerted by microglia. Nevertheless, this study did not employ direct approaches like ChIP to confirm C/EBPβ binding to the MMP9 promoter. Furthermore, the experiments relied solely on microglial monocultures and omitted astrocytes. This model may not fully capture the complex cellular interactions present in the brain.

In AD models, 15d-PGJ2 can be administered intranasally to improve cognitive function and glucose metabolism in brain tissue, and it can inhibit Aβ aggregation [36,37]. Researchers have recently developed magnetic nanocarriers for 15d-PGJ2 delivery. These carriers aim to enhance its targeting and anti-inflammatory effects in intracerebral hemorrhage, thus offering new clinical translation opportunities [38]. However, its application in Mn neurotoxicity has not yet been reported. In our study, prophylactic intranasal administration of 15d-PGJ2 increased the levels of this mediator in the striatum of Mn-exposed mice, while simultaneously inhibiting C/EBPβ activation and MMP9 expression, alleviating oxidative stress and microglial polarization, and improving cognitive behavior. Transcriptomic analysis identified genes whose expression was significantly reversed after 15d-PGJ2 intervention. These genes were mainly enriched in pathways related to inflammatory responses, oxidative stress, apoptosis, chemokine signaling, and neurodevelopment and function. The data from this study suggest that C/EBPβ may be one of its target molecules. However, this interpretation needs further testing, which can be done using PPARγ antagonists or gene knockout approaches. Furthermore, this study used a prophylactic dosing regimen, so therapeutic dosing effects were not evaluated. In addition, no pharmacokinetic data were collected. Consequently, the time to peak concentration and the steady-state brain levels of 15d-PGJ2 remain unclear.

Future research could take several directions. Prospective cohort studies would help validate whether serum 15d-PGJ2 and MMP9 can serve as useful biomarkers. These studies should also control for additional confounding factors. The transcriptional regulation of MMP9 by C/EBPβ needs clarification, and techniques such as ChIP and luciferase reporter assays could be applied for this purpose. Likewise, the receptors mediating 15d-PGJ2 actions remain unidentified, representing a mechanistic gap that requires further investigation. While 15d-PGJ2 is a known PPARγ ligand, we cannot exclude the possibility that its protective effects are mediated through PPARγ-independent pathways, such as direct modulation of NF-κB or C/EBPβ activity. Likewise, the receptors mediating 15d-PGJ2 actions remain unidentified. PPARγ antagonists or conditional knockout mice could be employed to address this question. The co-culture system models paracrine effects but is limited in mimicking the brain microenvironment. It should be noted that the present study used a prophylactic dosing regimen, which does not address whether 15d-PGJ2 can reverse existing pathology. However, in the context of occupational Mn exposure, where workers are exposed chronically over years, a preventive strategy carries practical relevance. Therapeutic dosing remains an important next step for future investigation. It should be noted that 15d-PGJ2 exhibits dose-dependent dual effects. At low concentrations, it acts as an anti-inflammatory and cytoprotective mediator, while higher concentrations may induce cytotoxicity through electrophilic modification of thiol groups. In the present study, the in vitro dose (5 μmol/L) was validated by CCK-8 assays showing no detectable cytotoxicity. The in vivo dose (300 ng/mouse, intranasal) was adopted based on previous research. Under both conditions, 15d-PGJ2 administration consistently reduced oxidative stress and inflammatory responses without observable toxicity. Nevertheless, we acknowledge the lack of systematic dose-finding for the in vivo dose as a limitation of this study. In addition, only male mice were used, and the generalizability of our findings to females remains to be determined. On the therapeutic side, the dosing regimen for 15d-PGJ2 should be optimized. Therapeutic dosing and pharmacokinetic-guided adjustments are both worth exploring, and structural analogs or nanocarrier systems might enhance stability. Although the current findings do not yet have direct clinical implications, they offer preliminary clues and point towards potential strategies for early warning and intervention in Mn-related cognitive impairment. Nevertheless, long-term efficacy and safety will need further investigation.

## 5. Conclusions

This study combines animal experiments with cellular experiments to investigate the relationship between Mn exposure and cognitive impairment, with a focus on changes in 15d-PGJ2.

In exposed workers and Mn-treated mice, Mn exposure was associated with reduced levels of 15d-PGJ2 and decreased expression of its synthase, L-PGDS. Bioinformatics analysis suggests that C/EBPβ and MMP9 are potential downstream molecules. In Mn-treated mice and BV2 cells, C/EBPβ activation was enhanced and MMP9 levels were elevated, accompanied by M1 polarization of microglia and increased ROS levels.

Knockdown of MMP9 or C/EBPβ attenuated microglial activation and protected neurons in co-culture. Exogenous 15d-PGJ2 reversed Mn-induced upregulation of both C/EBPβ and MMP9. It further lowered oxidative stress and pro-inflammatory markers. In addition, it improved cognitive behavior in Mn-exposed mice. Transcriptomic analysis showed that after 15d-PGJ2 treatment, the gene expression changes elicited by Mn tended to return to baseline. This reversal was most prominent in inflammatory and neurodevelopmental pathways. Knocking down Ptgds, the gene encoding L-PGDS, decreased 15d-PGJ2 levels and increased MMP9 levels. These alterations could be normalized by exogenous 15d-PGJ2.

In summary, decreased levels of 15d-PGJ2 and L-PGDS, along with elevated levels of C/EBPβ and MMP9, may contribute to Mn-induced neuroinflammation. Exogenous 15d-PGJ2 shows protective potential. However, further investigations are needed to establish direct causality and clinical applicability.

## Data Availability

The data presented in this study are available on reasonable request from the corresponding author.

## Supplementary Materials

The following supporting information can be downloaded at: https://www.mdpi.com/article/10.3390/antiox15080957/s1, Table S1. Primer sequences for the genes. Table S2. RNA-seq read counts and quality metrics for each sample. Table S3-1. Rotarod test results (time to fall, seconds). Table S3-2. Y-maze test results (spontaneous alternation, %). Table S3-3. Barnes maze test results (escape latency, seconds). Figure S1. FDR-adjusted differential expression analysis of Mn exposure and 15d-PGJ2 intervention.
