# Supplementary material for: Downregulation of the L-PGDS/15d-PGJ2 Pathway Is Associated with Manganese-Induced Neuroinflammation and Cognitive Impairment Involving C/EBPβ and MMP9
Source: Antioxidants (Basel). 2026 Jul 31;15(8):957. doi: 10.3390/antiox15080957 (PMC13510188; doi:10.3390/antiox15080957)
Supplement: Supplementary file 1 [file antioxidants-15-00957-s001.zip › antioxidants-4405785-supplementary.pdf]

Table S1. The sequence of primers for genes.

Species	Gene Name	Assession Number	Forward primer（5′→3′）	Reverse primer（5′→3′）	Product Length
mouse	*Gapdh*	NM_001411843.1	AATGTGTCCGTCGTGGATCTGA	GATGCCTGCTTCACCACCTTCT	83
mouse	*Arg1*	NM_007482.3	GATTATCGGAGCGCCTTTCT	CCACACTGACTCTTCCATTCTT	260
mouse	*CD206*	NM_008625.2	CAACAACTCCTGAACCCAAATG	AGCTTCCACTGCTCGTAATC	210
mouse	*Cebpb*	NM_001287738.1	CCGCCTTTAGACCCATGGAA	GCAGTCGGGCTCGTAGTAGA	50
mouse	*Mmp9*	NM_013599.5	CCTCCAACCTCACGGACAC	TTGGAATCGACCCACGTCTG	218
mouse	*Tnf*	NM_001278601.1	CCACCACGCTCTTCTGTCTAC	AGGGTCTGGGCCATAGAACT	103
mouse	*IL-1β*	NM_008361.4	TGTGAAATGCCACCTTTTGA	GGTCAAAGGTTTGGAAGCAG	94
human	*GAPDH*	NM_001357943.2	AGCCACATCGCTCAGACA	GCCCAATACGACCAAATCC	66
human	*PTGDS*	NM_000954.6	CATGTGCAAGTCTGTGGTG	ATGGTTCGGGTCTCACACTG	93

Table S2. RNA-seq read counts and quality per sample.

Sample	Raw Reads	Valid Reads	Valid Ratio(reads)	Q30%
Control_1	41108556	39502046	96.09	98.80
Control_2	44777662	43094464	96.24	98.84
Control_3	39475806	37977556	96.20	98.81
Mn_1	45356774	43673696	96.29	98.84
Mn_2	44597644	42889638	96.17	98.85
Mn_3	42787834	41295610	96.51	98.91
Treat_1	44163664	42480088	96.19	98.79
Treat_2	42837116	41172288	96.11	98.82
Treat_3	39396032	37777046	95.89	98.85

Note: Control, Mn, and Treat represent the control, manganese-exposed, and 15d-PGJ2 intervention groups, respectively, with three biological replicates per group (_1, _2, _3). Valid

Ratio indicates the proportion of reads passing quality filtering, and Q30 represents the percentage of bases with a quality score ⩾ 30 (i.e., ⩾99.9% base-calling accuracy).

Table S3-1. Rotarod test (time taken to fall, seconds).

Mouse	Con	15d-PGJ2	Mn	Mn+15d-PGJ2
1	187.1	176.2	94.4	137.8
2	167.3	145.2	82.8	132.3
3	167	138.5	78.9	114.1
4	147.9	127.7	73.8	106.5
5	134.9	120.5	64.6	101.3
6	124.2	94.2	61.1	92.1

Note: n = 6 mice per group. Data are from independent cohorts for each behavioral test.

Table S3-2. Y-maze test (spontaneous alternation, %).

Mouse	Con	15d-PGJ2	Mn	Mn+15d-PGJ2
1	37.50	38.89	21.05	27.27
2	34.62	33.333	23.08	27.27
3	33.33	33.33	23.81	28.57
4	33.33	33.33	25	30.00
5	26.92	30.00	26.09	30.77
6	26.32	25.00	18.75	33.33

Note: n = 6 mice per group. Data are from independent cohorts for each behavioral test.

Table S3-3. Barnes maze test (escape latency, seconds).

Mouse	Con	15d-PGJ2	Mn	Mn+15d-PGJ2
1	19.17	20.96	300.02	236.02
2	25.76	25.09	300.02	212.15
3	26.42	32.82	262.49	72.62
4	34.09	34.83	232.9	92.09
5	18.22	48.76	173.23	113.37
6	12.29	61.77	135.23	142.03

Note: n = 6 mice per group. Cut-off time was 300 seconds. Data are from independent cohorts for each behavioral test.

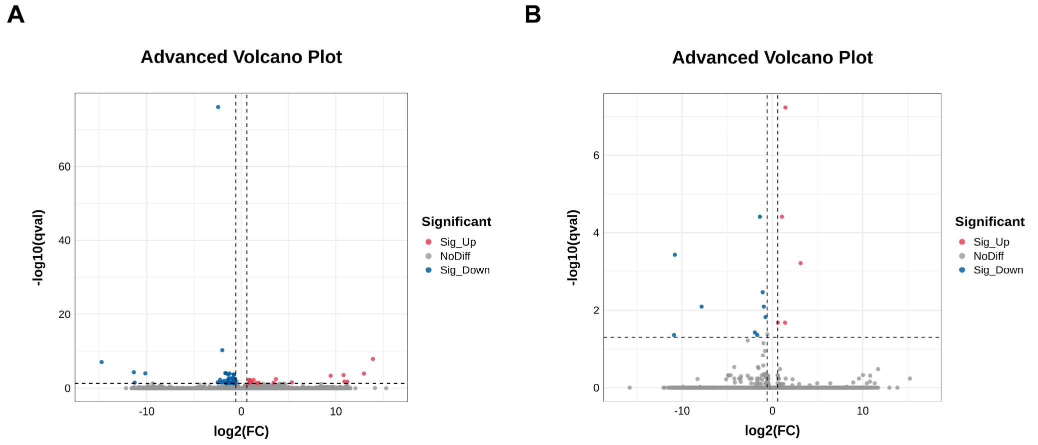

Figure S1. FDR-adjusted differential expression analysis for Mn exposure and 15d-PGJ2 intervention.

(A) Volcano plot showing differentially expressed genes in the striatum of Mn-exposed mice compared to controls (FC > 1.5, q < 0.05). Red and blue dots indicate significantly upregulated and downregulated genes, respectively. (B) Volcano plot showing differentially expressed genes in Mn-exposed mice with 15d-PGJ2 intervention compared to Mn-exposed mice alone (FC > 1.5, q < 0.05). FDR adjustment was performed using the Benjamini-Hochberg method. The main volcano plot (Figure 10B) and GO enrichment analysis in the main text were performed using FC > 1.2, $P < 0.05$ to ensure sufficient gene numbers for pathway detection.
